# RNAvigate: efficient exploration of RNA chemical probing datasets

**DOI:** 10.1093/nar/gkae089

**Published:** 2024-02-13

**Authors:** Patrick S Irving, Kevin M Weeks

**Affiliations:** Department of Chemistry, University of North Carolina, Chapel Hill, NC 27599-3290, USA; Department of Chemistry, University of North Carolina, Chapel Hill, NC 27599-3290, USA

## Abstract

Chemical probing technologies enable high-throughput examination of diverse structural features of RNA, including local nucleotide flexibility, RNA secondary structure, protein and ligand binding, through-space interaction networks, and multistate structural ensembles. Deep understanding of RNA structure–function relationships typically requires evaluating a system under structure- and function-altering conditions, linking these data with additional information, and visualizing multilayered relationships. Current platforms lack the broad accessibility, flexibility and efficiency needed to iterate on integrative analyses of these diverse, complex data. Here, we share the RNA visualization and graphical analysis toolset RNAvigate, a straightforward and flexible Python library that automatically parses 21 standard file formats (primary sequence annotations, per- and internucleotide data, and secondary and tertiary structures) and outputs 18 plot types. RNAvigate enables efficient exploration of nuanced relationships between multiple layers of RNA structure information and across multiple experimental conditions. Compatibility with Jupyter notebooks enables nonburdensome, reproducible, transparent and organized sharing of multistep analyses and data visualization strategies. RNAvigate simplifies and accelerates discovery and characterization of RNA-centric functions in biology.

## Graphical Abstract

**Graphical Abstract:**
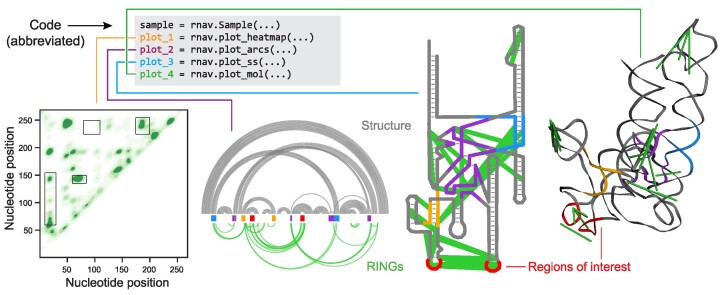

## Introduction

Primary, secondary, tertiary and quaternary structural features all affect RNA function. Primary structure, the nucleotide sequence of an RNA transcript, is determined by transcription start and termination sites, splice sites and polyadenylation sites. RNAs can contain diverse post-transcriptional modifications, which expand the primary sequence alphabet. Secondary structure is the pattern of both canonical and noncanonical base pairing and can involve stable helical regions, local pseudoknots and conformationally flexible regions. Tertiary structure, the three-dimensional arrangement of atoms in an RNA transcript, involves through-space interactions between nucleotide groups and can be stabilized by binding of ions, small molecules or macromolecules. Quaternary structure, the multimolecular structure of an RNA, includes its protein and RNA partner interactions.

These diverse levels of RNA structure can be interrogated with a large and expanding high-throughput toolbox (Supplementary Table S1). Primary sequence features can be determined by sequencing-based methods that measure transcript complexity (1) and numerous post-transcriptional modifications (2). Computational methods that model RNA secondary structure can (and generally should) be augmented by incorporating data from structure probing experiments. Current high-information structure probing methods employ structure-specific chemical probes that form covalent adducts with RNA. These covalent adducts are encoded into complementary DNA via reverse transcription as either mutations (mutational profiling or MaP) or reverse transcription stops and are quantified, per nucleotide, using high-throughput sequencing (3,4). Single-molecule correlated chemical probing (smCCP) strategies take advantage of the ability of MaP technology to detect multiple (potentially correlated) chemical events per molecule and provide deep insights into RNA secondary and tertiary structures, coordinated networks of protein binding and conformational ensembles (5). Proximity cross-linking methods use photo- or chemical cross-linking to characterize RNA inter- and intramolecular interactions and RNA–protein binding (6–8). These experimental and computational methods are powerful tools for modeling higher-order RNA structure and macromolecular complexes (9–11).

These strategies produce rich and complex data that pose two broad challenges for data exploration and hypothesis generation. First, to identify and highlight key features of a dataset, it is important to explore filtering and preprocessing steps, including background correction and normalization. Second, to gain a holistic understanding of an RNA system, multiple layers of data must be visualized together, in combination and as a function of experimental conditions. Many solutions exist for examining individual aspects of RNA structure, but a flexible and easy-to-use toolset that enables integration of multiple complex datasets, visualization in the formats commonly used by the RNA community, and efficient generation of biological hypotheses has been missing.

Jupyter notebooks are open-source interactive computing documents for use in web browsers and integrated development environments (12). These notebooks enable melding of code, analyses, visualizations and observational notes. A single document thus creates an environment for data exploration and hypothesis generation, a documented report of analysis findings, and a shareable and reproducible analysis pipeline. The widespread use and popularity of Jupyter notebooks has resulted in a well-developed ecosystem of open-source tools that support collaboration on and publication of data analyses. Until now, however, creation of the types of plots used by the RNA community required fluency in one of the programming languages compatible with Jupyter notebooks.

Here, we share a Jupyter-compatible Python module, RNAvigate, which simplifies creation of RNA community-standard visualizations and analyses derived from diverse data sources. RNAvigate rapidly aligns sequences and enables comparison, filtering, analysis and display of RNA structural information. RNAvigate is fast: Most operations are completed within seconds on modern, mid-tier CPUs. These features significantly streamline the analysis of complex RNA-centric datasets and the generation of impactful hypotheses regarding RNA structure–function interrelationships.

## Materials and methods

Data files and Jupyter notebooks containing all data and methods described here are included in the supplementary data and are available on GitHub (https://github.com/Weeks-UNC/RNAvigate_figures). Notebooks on GitHub were made interactive using the free Binder web service (part of the Jupyter Project). Users can reproduce these analyses and explore the data using RNAvigate in a web browser with no required downloads or software installations.

## Results and discussion

### RNAvigate scope

RNAvigate is a highly flexible data visualization and communication tool. Initial aligning and processing of structure probing data, typically a resource-intensive task, is performed, prior to using RNAvigate, with specialized software written and optimized for a specific purpose; for example, ShapeMapper (13,14) or RNA Framework (15) to analyze per-nucleotide reactivities or DanceMapper, DREEM or DRACO (16–18) to analyze smCCP experiments. The raw information provided by these programs is typically insufficient to understand a particular RNA system deeply. Indeed, the work of interpreting and developing hypotheses based on large-scale chemical probing data has just begun.

Individual chemical probing experiments are most advantageously interpreted in context with additional information, including measures of global structure, models of secondary and tertiary structures for individual RNA motifs, estimates of model confidence, and relationships to other RNA-centric features such as regulatory elements and protein-binding sites. RNAvigate accepts initial information from a wide variety of state-of-the-art technologies (and is extensible to others) and then facilitates integrative analysis of multiple classes of RNA-centric information and modeling.

We have found that researchers who are new to programming quickly learn to use RNAvigate to better understand diverse RNA systems and to produce intuitive visualizations of complex data, tasks that previously required programming expertise. RNAvigate is an accessible solution to efficiently produce illustrations that have proven especially impactful within the RNA structure community for generating mechanistic models and biological hypotheses.

### RNAvigate workflow

**Figure 1.**
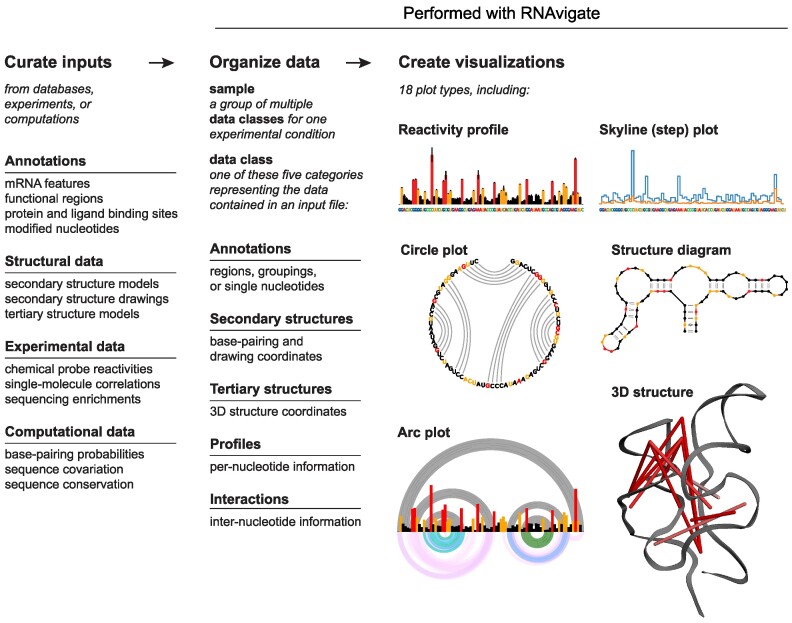
Workflow for data exploration using RNAvigate.

The RNAvigate workflow is organized into three steps (Figure 1). First, inputs are curated from the raw output of experiments, computations and database searches. Second, data classes are created and organized within units called samples by providing RNAvigate with input file names. Finally, visualizations are created using plotting functions built into RNAvigate.

RNAvigate includes a high-level interface, described here, that allows the second and third steps in this workflow to be executed using a single command each. The commands are designed to be simple to use, to automate many otherwise tedious tasks and to remain highly flexible. This design choice allows users to access RNAvigate features without learning object-oriented programming. RNAvigate also includes a more granular and extensive object-oriented programming interface that is not discussed in detail here. The full API is documented online (see the ‘Data availability’ section). This two-tiered design makes RNAvigate useful to both RNA bench scientists (including those with limited programming experience) and software and data specialists.

### Curate inputs

RNAvigate automatically parses RNA structural information in many standard file and data formats. The simplest inputs are annotations of the primary sequence of RNA. These annotations describe transcript features (translation start sites, exon junctions, untranslated regions), functional regions, modified nucleotides or binding sites for proteins, ligands and other nucleic acids. RNAvigate also accepts file formats output by structure modeling software [including RNAstructure and ViennaRNA (19,20)] and secondary structure drawing software [XRNA, VARNA, StructureEditor, FORNA and R2DT (19,21–23)]. Supported tertiary structure formats include CIF and PDB. Inputted experimental data can include chemical probe reactivities, such as those from MaP experiments, single-molecule events detected by smCCP, and sequencing enrichments such as those obtained from CLIP experiments. Inputted computational data can include pairing probabilities, covariation and sequence conservation. Most widely used file formats are natively supported by RNAvigate, and additional formats can be supported with simple code additions (Supplementary Table S1).

RNAvigate is primarily designed for analyzing single transcripts. However, RNAvigate can also read genome sequences (fasta), transcript annotations (GFF, GTF), and genomic or transcriptomic per-nucleotide or region data (BED/NarrowPeak or WIG). If provided with transcript identifiers, such as ENSEMBL or RefSeq IDs, RNAvigate extracts data in transcript coordinates. This information can include sequence, exon junctions, coding and untranslated regions, per-nucleotide measurements, protein-binding sites (eCLIP), and other annotations.

### Organize data: samples and data classes

Input data are stored in RNAvigate as one of five data classes: annotations, secondary structures, tertiary structures, profiles or interactions (Figure 1). Data classes standardize data representation for diverse input file formats and enable RNAvigate functionality. A sample in RNAvigate is an organizational grouping of multiple data classes (e.g. experimental data, structure models and sequence annotations) describing a single RNA interrogated under a specific experimental condition. The user creates a sample and populates it with inputs, which RNAvigate converts to data classes. This step is repeated to accommodate multiple experimental conditions, standardize data organization across conditions, and simplify downstream commands.

Annotations contain regions of interest, single and grouped nucleotides of interest, and primer-binding sites. Secondary structures contain base-pairing information and optional diagram-drawing coordinates for secondary structure models. Tertiary structures contain atomic coordinates for tertiary structure models. Profiles contain any type of experimental or computational measurement made on a *per-nucleotide* basis. Examples of per-nucleotide data include chemical probing reactivities, structure-based entropies and sequence conservation. Interactions contain experimental or computational measurements made *between* two nucleotides. These measurements include sequence covariation, base-pairing probabilities, single-molecule correlations and cross-linking information. These five classes of transcript-centric information are flexible and extensible and allow RNAvigate to represent most types of RNA structural data (see Supplementary Table S1).

### Create visualizations

**Figure 2.**
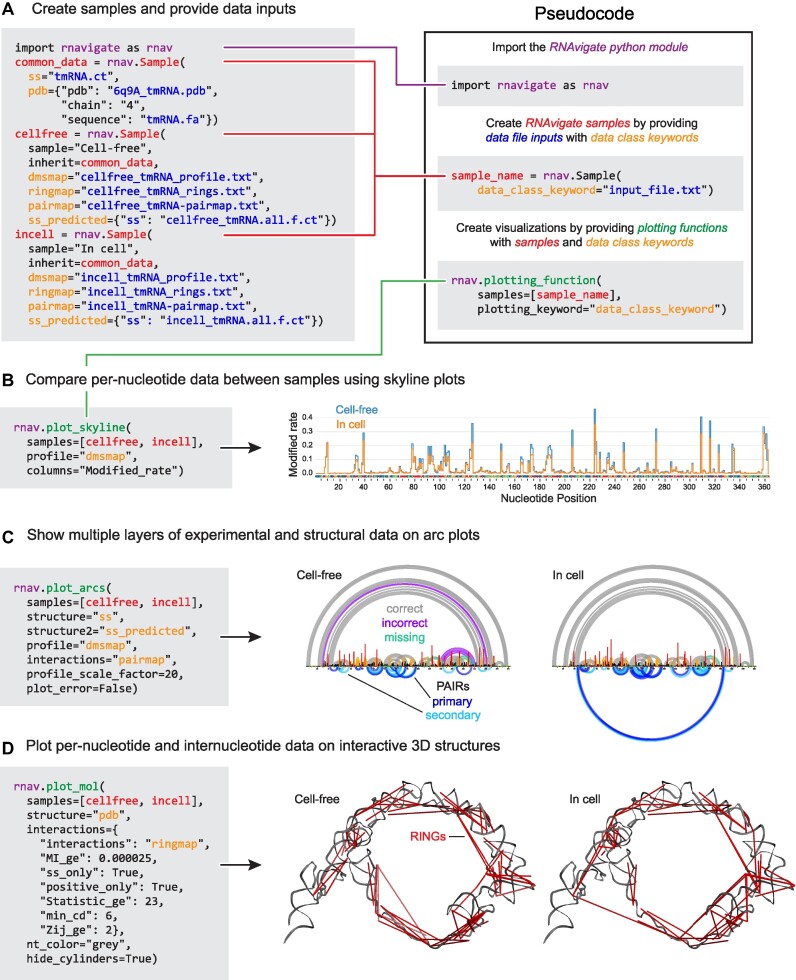
A complete RNAvigate workflow: smCCP of transfer–messenger RNA (tmRNA). In this example, full RNAvigate code is shown and essential coding steps are explained with code-like examples. (**A**) First, the RNAvigate module is imported and then in-cell and cell-free dimethyl sulfate (DMS) probing data and contextual structure information are imported. These steps create RNAvigate samples, each containing all of the data related to a specific experimental condition. (**B**) To compare data between experimental conditions, a skyline plot was generated. (**C**) To show multiple types of data on single plots, two arc plots with the literature-accepted base pairs, data-informed minimum free energy (MFE) structure predictions, and PAIRs (pairing ascertained from interacting RNA strands) were generated. Base pairs are labeled ‘correct’ (gray) if predicted and in the accepted structure, ‘incorrect’ (purple) if predicted and not in the accepted structure, and ‘missing’ (green) if not predicted and in the accepted structure. PAIRs of the highest confidence are labeled ‘primary’ (dark blue), otherwise are ‘secondary’ (light blue). (**D**) RINGs (RNA interacting groups; red) from cell-free and in-cell experiments were then plotted on the three-dimensional tmRNA backbone (gray) obtained from the cryo-electron microscopy (cryo-EM) structure. Data are from (28).

RNAvigate automates many community-standard analyses and visualizations with easy-to-use, flexible functions. Two standard visualizations commonly used by the RNA community are per-nucleotide graphs and connectivity diagrams. Per-nucleotide graphs display nucleotide position along the *x*-axis and a measured value on the *y*-axis. RNAvigate can output two types of per-nucleotide graphs: colored bar graphs for displaying a single dataset (Figure 1) and stepped-line graphs (skyline plots) for comparing multiple datasets (Figures 1 and 2B).

RNAvigate can create four types of connectivity diagrams: arc plots, circle plots, secondary structure diagrams and interactive three-dimensional molecular renderings. Connectivity diagrams display through-space intranucleotide interactions such as base pairs, cross-links or correlated single-molecule events. Arc plots arrange nucleotides along the *x*-axis and show interactions as semicircles (Figures 1, 2C, 5A and 6C). Circle plots arrange nucleotides around a circle with interactions drawn as parabolas (Figure 1). Secondary structure diagrams have custom arrangements of nucleotides and display interactions as lines (Figures 1, 4A and B, and 5B). Finally, interactive three-dimensional molecule renderings arrange nucleotides according to atomic coordinates and display interactions as thin cylinders (Figures 1, 2D, 4C and 5C). Other visualizations automated by RNAvigate include 3D distance histograms, heatmaps (Figure 4B) and contour plots (8,24). Automated analyses include identification of regions with low SHAPE reactivity and low Shannon entropy (lowSS regions) (25) (Figure 2), linear regressions (Figure 6A), deltaSHAPE (26) and windowed area under receiver operating characteristic curves (27). Users can view those plotting functions that are compatible with a given sample with the rnav.plot_options() function.

### Example workflows

**Figure 3.**
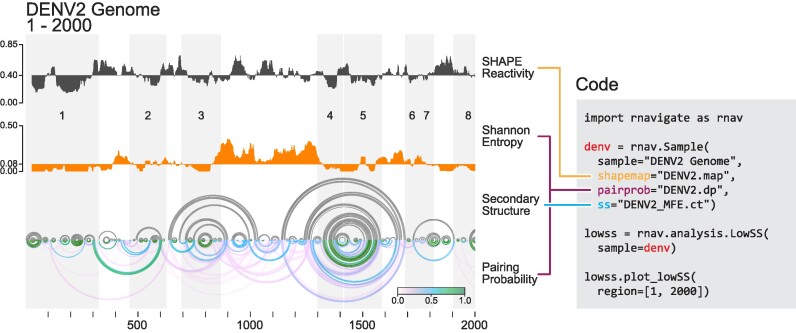
Identification and visualization of lowSS regions in the dengue virus serotype 2 (DENV2) genome using RNAvigate. Median (top) SHAPE reactivity and (middle) Shannon entropies over 51-nucleotide windows across the first 2,000 nucleotides of DENV2 genome. (Bottom) Modeled MFE structure (gray) and pairing probabilities (see scale) illustrated as arc plots. Plots share the same *x*-axis (nucleotide position) for straightforward comparison. Regions with median SHAPE reactivity below 0.4 and median Shannon entropy below 0.15 are defined as lowSS regions and are shaded in gray and numbered. Full RNAvigate code used to generate this analysis and visualization is shown at right; data are from (25).

**Figure 4.**
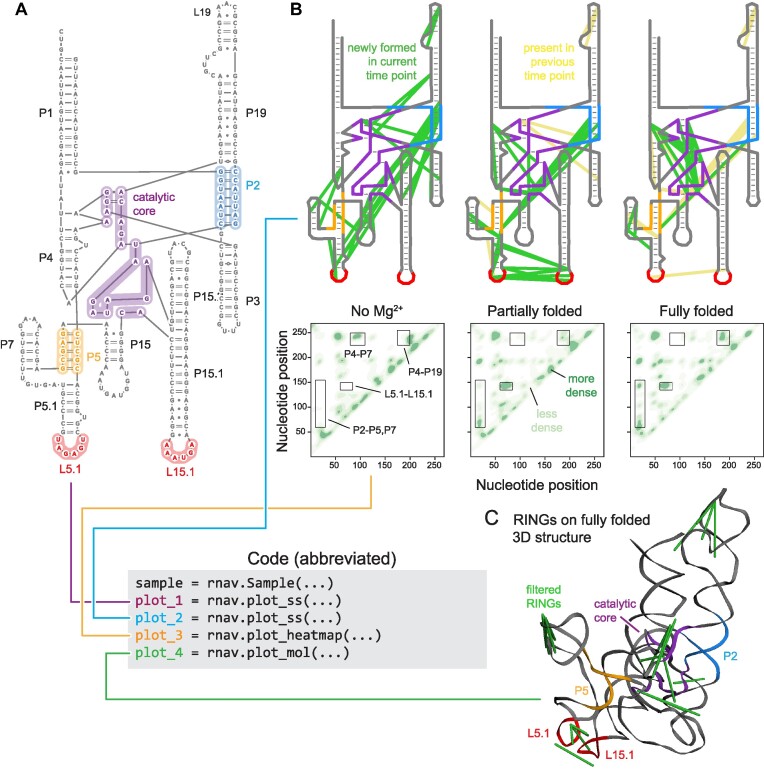
Time-resolved probing and folding of the ribonuclease P (RNase P) RNA, visualized using RNAvigate. (**A**) Secondary structure of the catalytic core of RNase P RNA. Regions involved in major time-dependent changes are highlighted. (**B**) (Top) Through-space RINGs as a function of folding time superimposed on the secondary structure. Newly formed RINGs at each time point are shown in green; RINGs retained from the prior time point are yellow. (Bottom) Heatmaps of RING densities at each time point. (**C**) Three-dimensional structure of the RNase P RNA with superimposed RINGs (green). Data are from (34).

**Figure 5.**
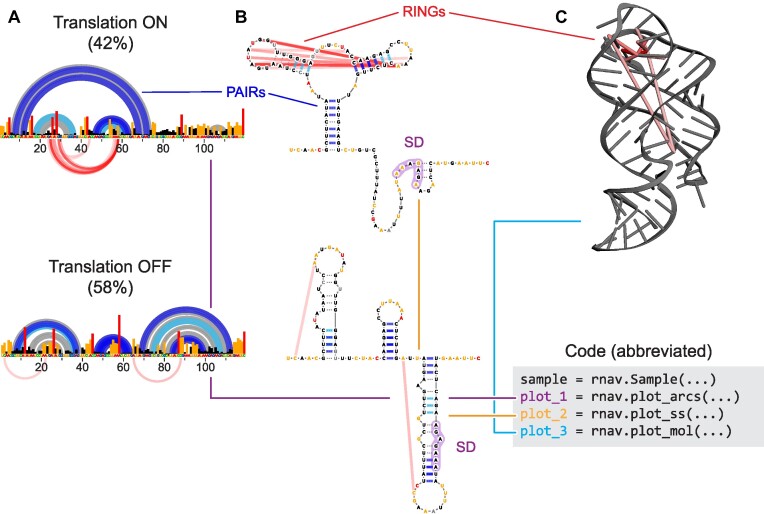
DANCE-MaP analysis of the *add* riboswitch ensemble visualized using RNAvigate. (**A**) PAIRs, RINGs (each shown as arc plots), and reactivity profiles for DANCE-identified ON and OFF states. (**B**) Secondary structure drawings of ON and OFF states with PAIRs (blue) and RINGs (red) superimposed. The Shine–Dalgarno sequence (SD) is highlighted. (**C**) Structure of *add* riboswitch in the ON state with RINGs unique to this state shown. Data were obtained in the absence of adenine ligand but, nonetheless, show that the *add* riboswitch populates the ON state. Data are from (16).

**Figure 6.**
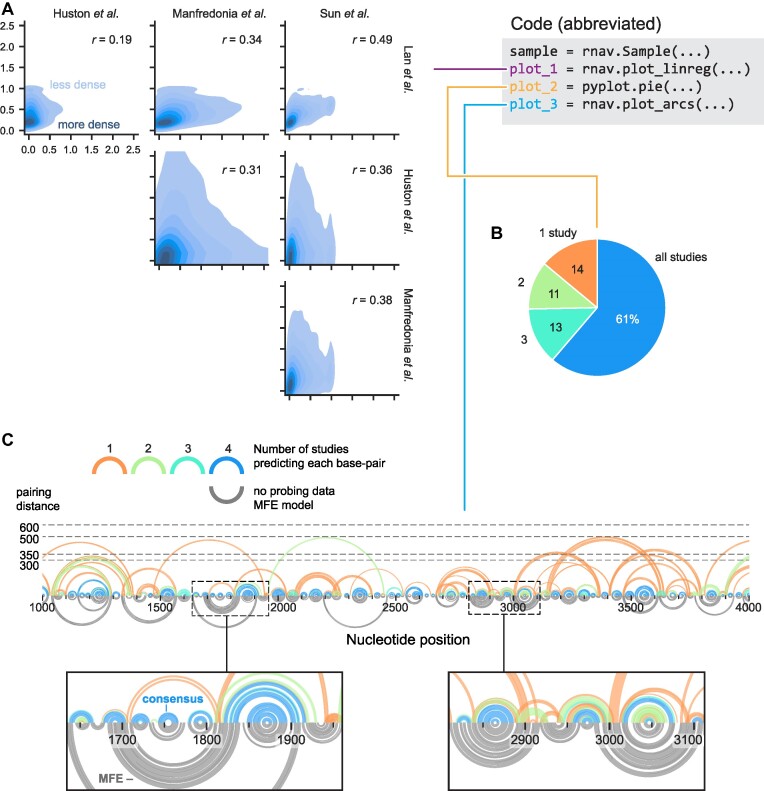
Comparative analysis of SARS-CoV-2 structure probing data and secondary structure models across four studies. (**A**) Study versus study per-nucleotide reactivities (shown as kernel density estimates) and Pearson correlations from linear regression analyses. (**B**) Pie chart showing percentage of predicted base pairs observed across the four studies. (**C**) Visualization of modeled base pairs as a function of study consensus (upper arcs) compared to the MFE structure (gray arcs). All predicted base pairs from all studies are shown (as arcs); colors indicate the number of studies predicting that base pair. Dotted lines indicate maximum pairing distance constraints used by each study (modeled pairing arcs will not exceed this line). Analyses shown here correspond to 7000 nucleotides of ORF1ab (positions 266–7265); data for this region were available for all studies. Data are from (27,38–40).

The following sections provide examples of the use of RNAvigate to analyze RNA structure and to generate hypotheses regarding underlying RNA-mediated function. Figures 3–5 replicate published figures and illustrate the ability of RNAvigate to create high-content illustrations efficiently. Figure 6 compares data from four independent studies and illustrates the flexibility of RNAvigate in integrating analysis of data from multiple sources, in different original formats. The raw data, full code and Jupyter notebooks used to generate these analyses and figures are fully and freely available via GitHub and Binder; the latter allows interaction with these notebooks with no required software download or installation (see the ‘Materials and methods’ section).

### smCCP analysis

The development of smCCP is a revolution in RNA structure probing as these strategies allow multiple classes of through-space interactions to be measured in a simple chemical probing experiment (5). smCCP measures structural communication between two nucleotides as correlated chemical modification events (5). Correlated events from probing experiments can be analyzed and attributed to base-pairing interactions (PAIRs) or to through-space interactions reflective of tertiary structure (RINGs). Whereas smCCP experiments are generally straightforward to perform, their analysis and interpretation can be complex. RNAvigate dramatically streamlines the required analysis. Here, we illustrate one such analysis, showing all RNAvigate code.

Bacterial tmRNA is responsible for rescuing stalled ribosomes. This RNA has a complex secondary structure, including four pseudoknots, and a large open ring tertiary structure. Secondary structures for tmRNAs are poorly predicted by computational methods alone (28). We used RNAvigate to facilitate analysis of publicly available structure probing data of *Escherichia coli* RNA treated under in-cell and cell-free conditions using DMS (28). The original dataset was used to assess the ability of PAIRs to improve modeling of RNA secondary structure in cells. We confirmed that result, and further explored through-space tertiary interactions measurable from these data but not previously reported.

We collected raw sequencing reads from the DMS probing experiments from a sequence read archive, the literature-accepted secondary structure in the form of a .ct file and the cryo-EM structure from the RCSB (29). Raw sequencing reads were analyzed with ShapeMapper2, PairMapper and RingMapper (13,28). ShapeMapper2 calculates per-nucleotide mutation rates reflective of DMS reactivity with RNA, PairMapper detects smCCP events indicative of base pairing (PAIRs) and RingMapper detects smCCP events arising from through-space interactions (RINGs). DMS reactivities and PAIRs were used to direct minimum free energy (MFE)-based secondary structure modeling using the RNAstructure fold program (19) (using -dmsnt and -x parameters, respectively).

The RNAvigate Python module was imported into a Jupyter notebook and given the alias rnav (Figure 2A). Input file names were provided for each experimental condition using rnav.Sample(). This important step accomplishes three tasks simultaneously: The data contained in each file are associated with an RNAvigate sample, converted into a data class, and assigned to a short keyword for easy access. Here, each RNAvigate sample contains all data relating to a specific experimental condition: cell-free or in-cell. These RNAvigate samples were assigned to variable names cellfree and incell, respectively (Figure 2A). Three classes of smCCP data analysis were performed; each plot was created with a single command, optionally with modifying arguments.

We first visualized overall reactivity profiles under in-cell and cell-free conditions using a skyline plot (Figure 2B). Per-nucleotide reactivity rates were similar but slightly higher under cell-free conditions, reflective of a more highly structured state for this noncoding RNA in cells, a common observation. Next, an arc plot was used to compare modeled and accepted structures and to visualize the DMS reactivities and PAIRs that informed the structure model. This visualization revealed that in-cell data produced a more accurate model of the secondary structure than did data obtained under cell-free conditions. The RNAvigate analysis showed that the increased accuracy is driven by more abundant and accurate PAIRs (Figure 2C). Finally, RINGs were visualized superimposed on the cryo-EM structure. This superposition revealed extensive structural communication between the adjacent pseudoknots along the outer ring of the structure and an absence of structural communication across the circle-like opening (Figure 2D).

### Analysis of regions of low SHAPE reactivity and low Shannon entropy

One especially informative analysis of long RNAs is to identify regions that have both low SHAPE reactivities, indicative of extensive base pairing, and low Shannon entropy, which indicates a well-defined structure. These lowSS regions are strongly associated with functional regions in long RNAs (25,30,31). RNAvigate makes identification and visualization of lowSS regions straightforward. Here, we describe use of RNAvigate to reproduce a useful analysis based on a publicly available dataset obtained from SHAPE probing of DENV2 genomic RNA (25).

Dengue virus has a positive-sense, single-stranded RNA genome of ∼11 kb. Conserved structures in the 5′ and 3′ untranslated regions regulate viral processes and are well characterized (32), but identification of functional structures in less conserved regions has proven challenging. Previously reported analyses of lowSS regions made it possible to define novel regions of functional importance and to identify regions likely to have complex tertiary structures (25,33).

The RNAstructure programs fold and partition were used to model an MFE structure and determine base-pairing probabilities, respectively, using SHAPE reactivity data as pseudo-free-energy constraints (--shape parameter) (19). These results were imported into a Jupyter notebook with RNAvigate. RNAvigate was used to calculate 51-nucleotide windowed median SHAPE reactivities and median Shannon entropies based on the provided pairing probabilities (Figure 3, top and middle). The program then identified lowSS regions where the windowed median SHAPE reactivity was below 0.4 and the median Shannon entropy was below 0.15. RNAvigate enables visualization of this analysis as stacked profiles of SHAPE reactivity and Shannon entropy and of predicted MFE structures and pairing probabilities displayed as arc plots (Figure 3). Visualization of the first 2000 nucleotides of the DENV2 genome revealed eight lowSS regions.

This straightforward application of RNAvigate rapidly recapitulated the results of the published analysis (25), which required significantly greater effort to create. The original illustration was created using command-line tools to generate a PDF image of the arc plot and a spreadsheet program to calculate and to visualize windowed SHAPE and Shannon entropy profiles. These plots were copied into a vector drawing program, where they were manually adjusted to align the *x*-axes and to highlight lowSS regions. Plot labels, legends and axes were added manually. RNAvigate accomplished a similar, if not improved, result in three lines of code; similar efficiencies were achieved in all examples described here.

### Analysis of time-resolved probing

Many dynamic RNA processes are best analyzed in a time-resolved way. These experiments result in complex datasets across multiple experimental conditions. In a recent example of time-resolved analysis, trimethyloxonium (TMO) was devised as a fast-reacting alkylating reagent and used to study the folding of the catalytic core of the RNase P enzyme from *Bacillus stearothermophilus* (34). The catalytic core of RNase P is a long noncoding RNA that catalyzes cleavage of 5′ leader sequences of precursor transfer RNAs (tRNAs), an essential step for tRNA maturation. TMO reacts with RNA via a chemical mechanism similar to that of DMS, but the TMO reaction is 90 times faster. The *in vitro*-transcribed RNase P RNA was treated with TMO, initially in the absence of Mg^2+^, and then at multiple time points after adding Mg^2+^. Raw sequencing reads were obtained corresponding to MaP analyses at each time point. The folding pathway was examined in smCCP experiments and involved creating secondary and tertiary structure diagrams and heatmaps of through-space RINGs for every time point in the experiment. This complex analysis is now rendered straightforward with RNAvigate.

For the RNAvigate analysis, the raw data were analyzed using ShapeMapper2 and RingMapper. A secondary structure diagram and three-dimensional model of the RNA [based on PDB 3DHS (35)] were obtained from the authors. A detailed secondary structure was generated in RNAvigate that includes annotations of the catalytic core, the P2–P5 pseudoknot and the L5.1–L15.1 loop–loop interaction (Figure 4A). The strongest RINGs with positive correlations were displayed on the secondary structure drawing. This superposition revealed large changes in the structural communication network as folding progresses. Analysis of RINGs as a function of time revealed that the L5.1–L15.1 loop–loop interaction formed very quickly and was present in an early partially folded state. In the fully folded state, interactions within the catalytic core became more pronounced, revealing that this structure forms more slowly (Figure 4B, top).

The spatial density of positively correlated RINGs, plotted as heatmaps, across the three states revealed additional interactions where through-space RING correlations changed during the folding process (Figure 4B, bottom). This visualization confirmed rapid formation of the L5.1–L15.1 loop–loop interaction upon addition of Mg^2+^. These heatmaps also reveal that interactions in the P4–P7 region disappeared and interactions in P2–P5,P7 region formed quickly after addition of Mg^2+^, indicating interdependency between the pseudoknot helices. RING densities also revealed a slower forming interaction between P4 and P19. Finally, RINGs from the fully folded state were filtered to isolate likely tertiary interactions and plotted on the three-dimensional structure (Figure 4C). There is a high density of RINGs in the catalytic core, and this fully folded state closely matches the known structure (Figure 4C). This example illustrates that complex, time-resolved data are analyzed efficiently using RNAvigate.

### Deconvolution of a conformational ensemble

smCCP strategies can now resolve complex conformational ensembles of RNA, including in the cellular environment (5). The DANCE (deconvolution and annotation of ribonucleic conformational ensembles) framework uses machine learning to deconvolute smCCP data to define and to characterize the dominant states in an RNA conformational ensemble. This single experiment and subsequent analyses produce multiple layers of data for each state, including population percentages, reactivity profiles, through-space PAIRs and RINGs, secondary structure models and base-pairing probabilities (16). Using RNAvigate, analysis of this layered information, and closely related smCCP experiments (7,17,18,36), is performed efficiently.

We used RNAvigate to reanalyze a previous DANCE-MaP analysis of the adenine riboswitch (16). The adenine riboswitch region of the *Vibrio vulnificus add* messenger RNA (mRNA) populates two dominant conformations. Adenine binds to the ON state and shifts the equilibrium toward this state. Formation of the ON state results in production of adenine deaminase, because, in this conformation, the Shine–Dalgarno sequence of the mRNA is single-stranded and accessible to the ribosome. In the OFF state, the Shine–Dalgarno sequence is sequestered in a duplex. Conventional per-nucleotide structure probing methods measure population averages and are misleading for this riboswitch, because the RNA samples two well-populated conformations (16).

For RNAvigate analysis, data files from the DANCE-MaP analysis of the adenine riboswitch in the absence of adenine (16) were obtained from the Gene Expression Omnibus (GSE182552). These data files contained reactivity profiles, PAIRs and RINGs for each of the dominant conformations. The foldClusters module of DanceMapper was used to predict MFE structure models for each conformation from the reactivities and PAIR constraints (--bp parameter) for each conformation. These structure models were loaded into StructureEditor (19) and manually arranged to match the original publication. A three-dimensional structure for the translation ON state was obtained from the PDB (4TZX) (37). File names were then provided to RNAvigate within a Jupyter notebook and used to create the visualizations.

RNAvigate analysis showed that PAIRs accurately and sensitively detected most base-paired helices, recapitulating the accepted secondary structures of both states (Figure 5A). Consistent with the original publication (16), even in the absence of ligand, both ON and OFF states are significantly populated. RINGs captured the loop–loop tertiary interaction stabilizing the three-dimensional structure of the riboswitch ON state (Figure 5B and C). In the context of the crystal structure, through-space RING interactions link two close-in-space loops (Figure 5C). RNAvigate efficiently analyzes information-rich DANCE-MaP experiments and straightforwardly visualizes the multiple dominant states present in an RNA structural ensemble.

### Comparison of chemical probing methods

Comparing chemical probing datasets from different laboratories can inform understanding of the effects of experimental choices. Four groups recently reported secondary structure models of the SARS-CoV-2 RNA genome based on in-cell structure probing (27,38–40). We used RNAvigate to compare these per-nucleotide reactivities and secondary structure models, and to evaluate the overall robustness of chemical probing to produce consensus secondary structure models.

The SARS-CoV-2 betacoronavirus is encoded by a single-stranded, positive-sense RNA genome of ∼29 kb. The four studies examined here performed the same steps, but experimental and computational parameters differed at each step (detailed in Supplementary Table S2) (27,38–40). First, chemical probing (SHAPE or DMS) was performed on native viral RNA in infected cell cultures. Sites of adducts were then encoded using MaP or an RT-STOP strategy (using one of three reverse transcriptases). Finally, per-nucleotide reactivities were calculated and used to guide thermodynamic modeling of viral genomic RNA secondary structure.

Per-nucleotide reactivities and secondary structure model data files were downloaded from the supporting data for each publication. Downloaded data files were either in standard formats (.ct, dot-bracket, ShapeMapper2 profile.txt or RNA Framework XML), all natively supported by RNAvigate, or in Excel spreadsheets (loaded via the Python command Pandas.read_excel). We focused on population average reactivities and structure models in a representative region spanning the first 7 kb of ORF1ab.

Reactivity data were unintuitively dissimilar as indicated by Pearson correlation coefficients (0.19 < *r* < 0.49) and as visualized using kernel density plots (Figure 6A). Nevertheless, 61% of modeled base pairs were shared across all studies (Figure 6B). All four studies based their structure modeling strategy on the Δ*G*_SHAPE_ (or Δ*G*_DMS_) framework (4). In this strategy, structure probing reactivities are converted to Δ*G* bonuses and used to improve thermodynamics-based calculations of high-probability structures (4,19). The similarity in structural models supports previous findings that this structure modeling strategy is robust. Modeling large structures typically requires imposing a maximum pairing distance constraint to prevent over-modeling of long-range base pairs. The four studies chose different constraints, ranging from 300 to 600 nucleotides, limiting similarity in structure models, especially for longer-range interactions (Figure 6C). These differences can have important functional consequences (4,5) and can be resolved by smCCP (28).

The data-driven models differed notably from the MFE structure obtained using no probing data. We found that 24% of the 2257 total base pairs in the MFE model (calculated with maximum pairing distance of 300 base pairs) do not appear in any probing-directed model and that 12% of the 1234 consensus base pairs, defined as those present in models from at least three studies, are not present in the MFE model. Differences between the data-driven and MFE models are not evenly distributed; some regions are modeled particularly poorly, and it is not possible to define *a priori* where the MFE model is most misleading (Figure 6C, bottom). In sum, this exercise demonstrates that RNAvigate efficiently compares reactivities and structure models from multiple experiments, identifying both consensus and divergent helices, and emphasizes that there are many structural elements that are difficult to model without guiding per-nucleotide experimental information.

### Perspective

RNA structure–function interrelationships are complex. Powerful and orthogonal computational and experimental strategies have been developed to identify the hierarchical and interdependent elements of RNA structure. It is both important and challenging to understand, integrate and interpret these rich layers of information. The visualization and analysis tools of RNAvigate facilitate analysis of diverse structural features of RNA. RNAvigate in conjunction with Jupyter notebooks enables rapid analysis and thorough documentation of data quality control, data exploration, and hypothesis generation. RNAvigate then directly generates publication-quality figures.

RNAvigate, when used within a Jupyter notebook, provides a convenient, nonburdensome platform for documentation and for transparent and reproducible sharing, which enhances the longevity and impact of analyses of RNA experimental and computational data. Full pipelines for analysis and figure generation can be recreated, repurposed or reconfigured without the requirement to download additional data or install specialized software, as demonstrated by the Jupyter notebooks accompanying this manuscript.

RNAvigate is modular and readily extensible and thus provides a framework to support analysis and visualization of diverse experimental and computational data types. We anticipate that RNAvigate will enhance development, implementation, reproducibility and sharing of strategies for discovery and characterization of diverse RNA-centric functions in biology.

## Supplementary Material

gkae089_Supplemental_Files

## Data Availability

The RNAvigate Python module is available on GitHub (https://github.com/Weeks-UNC/RNAvigate) and is installable via Docker by following the instructions included in the documentation. RNAvigate documentation is available on Read the Docs (https://rnavigate.readthedocs.io).
