# Supplementary material for: RNAvigate: efficient exploration of RNA chemical probing datasets
Source: Nucleic Acids Res. 2024 Feb 13;52(5):2231–41. doi: 10.1093/nar/gkae089 (PMC10954457; doi:10.1093/nar/gkae089)
Supplement: gkae089_Supplemental_Files [file gkae089_supplemental_files.zip › RNAvigate_SI_static_notebooks.zip!/RNAvigate_SI_static_notebooks/F2_smCCP_tmRNA.html]

F2\_smCCP\_tmRNA


# smCCP analysis of tmRNA¶

This is an example RNAvigate workflow for visualization of smCCP analysis of tmRNA.

Data were originally published by Mustoe et. al. 2019. pdf

The supplemental data for this publication include processed ShapeMapper2 and PairMapper data, reference sequence and secondary structure of tmRNA, and DMS/PAIR-constrained structure models. To obtain RingMapper files, the raw sequencing reads were downloaded from the GEO (GSE135211), and processed with ShapeMapper v2.1.5 (using `--amplicon` and `--output-parsed-mutations` parameters) and Ringmapper (using default parameters). Cryo-EM structure was downloaded from the RCSB (6Q9A), and the tmRNA chain was isolated using pymol. These steps yield the files needed in this example:

- 6q9A\_tmRNA.pdb
- cellfree\_tmRNA\_profile.txt
- cellfree\_tmRNA\_rings.txt
- cellfree\_tmRNA-pairmap.txt
- cellfree\_tmRNA.all.f.ct
- incell\_tmRNA\_profile.txt
- incell\_tmRNA\_rings.txt
- incell\_tmRNA-pairmap.txt
- incell\_tmRNA.all.f.ct
- tmRNA.ct
- tmRNA.fa

## Import RNAvigate¶

In [ ]:

```
import rnavigate as rnav
```

## Define the experimental samples and provide input file names¶

- `sample`: an arbitrary string used as a label on plots
- `dmsmap`: a ShapeMapper2 profile.txt. Data will be renormalized for DMS by RNAvigate automatically.
- `ringmap`: a RingMapper correlations file
- `pairmap`: a PairMapper pairmap.txt file
- `ss`: a previously published structure as a .ct file
- `ss_predicted`: a data-informed minimum free energy model as a .ct file
- `pdb`: a cryo-EM structure model as a .pdb file

In [ ]:

```
common_data = rnav.Sample(
    sample="shared data",
    ss="tmRNA.ct",
    pdb={"pdb": "6q9A_tmRNA.pdb",
         "chain": "4",
         "sequence": "tmRNA.fa"})

cf = rnav.Sample(
    sample="Cell-free",
    inherit=common_data,
    dmsmap="cellfree_tmRNA_profile.txt",
    ringmap="cellfree_tmRNA_rings.txt",
    pairmap="cellfree_tmRNA-pairmap.txt",
    ss_predicted={"ss": "cellfree_tmRNA.all.f.ct"})

ic = rnav.Sample(
    sample="In cell",
    inherit=common_data,
    dmsmap="incell_tmRNA_profile.txt",
    ringmap="incell_tmRNA_rings.txt",
    pairmap="incell_tmRNA-pairmap.txt",
    ss_predicted={"ss": "incell_tmRNA.all.f.ct"})
```

## Compare modified mutation rates with a skyline plot¶

In [ ]:

```
plot = rnav.plot_skyline(
    samples=[cf, ic],
    profile="dmsmap",
    columns="Modified_rate")

# saves plot as an svg file
# plot.save("tmrna_skyline.svg")
```

## Compare structure models to previously published model and display PAIRs¶

In [ ]:

```
plot = rnav.plot_arcs(
    samples=[cf, ic],
    sequence="ss",
    structure="ss",
    structure2="ss_predicted",
    profile="dmsmap",
    interactions="pairmap",
    profile_scale_factor=20,
    plot_error=False
)

# save the plot as an svg file
# plot.save("tmrna_arcs.svg")
```

## Display filtered RINGs on a cryo-EM structure¶

Here, we calculate mutual information scores for RINGs to be used to filter RINGs.

See the Figure 4 notebook for more detail on this calculation.

In [ ]:

```
for sample in [cf, ic]:
    sample.data["ringmap"].data.eval("MI = Statistic / (2 * Mod_Depth)", inplace=True)

appearance_arguments={
    "width": 1000,
    "height": 1000,
    "background_alpha": 0,
    "orientation": [-177.55,-335.68,-210.36,-408.72,-0.27,-0.14,0.22,-0.93],
    "hide_cylinders": True}

plot = rnav.plot_mol(
    samples=[cf, ic],
    structure="pdb",
    colors="grey",
    interactions={
        'interactions': "ringmap",
        "MI_ge": 0.000025,
        "ss_only": True,
        "positive_only": True,
        "Statistic_ge": 23,
        "min_cd": 15,
        "Zij_ge": 2},
    # # appearance arguments
    # width=1000,
    # height=1000,
    # background_alpha=0,
    orientation=[-177.55,-335.68,-210.36,-408.72,-0.27,-0.14,0.22,-0.93],
    hide_cylinders=True
    )
```

You appear to be running in JupyterLab (or JavaScript failed to load for some other reason). You need to install the 3dmol extension:   
jupyter labextension install jupyterlab\_3dmol

In [ ]:

```
# plot.save()
```
