# Supplementary material for: RNAvigate: efficient exploration of RNA chemical probing datasets
Source: Nucleic Acids Res. 2024 Feb 13;52(5):2231–41. doi: 10.1093/nar/gkae089 (PMC10954457; doi:10.1093/nar/gkae089)
Supplement: gkae089_Supplemental_Files [file gkae089_supplemental_files.zip › RNAvigate_SI_static_notebooks.zip!/RNAvigate_SI_static_notebooks/F1_simple_plots.html]

F1\_simple\_plots


# Simple plotting examples with TPP riboswitch¶

The data used in this example was collected by a graduate student in our lab, David Qiu.
These data are used here to illustrate the types of plots that can be created using RNAvigate.

In breif, a DMS-MaP experiment was performed on an IVT construct of a TPP riboswitch.
Raw sequencing data were analyzed using ShapeMapper v2.1.5 (with `--amplicon` and `--output-parsed-mutations` parameters).
The resulting parsed mutation strings files (`_parsed.mut`) were analyzed further using RingMapper (default parameters).

ShapeMapper2 data were used with RNAStructure's partition (with `--dmsnt` parameter) and ProbabilityPlot (with `-t` parameter) to produce a pairing probabilities text file.
A crystal structure was obtained from the PDB (2GDI).
A secondary structure diagram, based on the crystal structure, was created manually using StructureEditor.

These steps yield the 5 data files used in this notebook:

- DMS\_TPP\_profile.txt
- DMS\_TPP\_rings.txt
- 2gdi.pdb
- TPP-2GDI.nsd
- TPP-dms-bp.dp

Links to pre-processing software:

- ShapeMapper2
- RingMapper
- RNAstructure and StructureEditor

## Import RNAvigate¶

In [ ]:

```
import rnavigate as rnav
```

## Define the experimental samples and provide input file names¶

This step creates RNAvigate data classes representing the data contained in input files.

- `sample`: an arbitrary string used as a label on plots
- `dmsmap`: a ShapeMapper2 profile.txt.
  - When using `dmsmap` instead of `shapemap` as the keyword, data are automatically rescaled using the same convention as PairMapper (manuscript citation 27).
- `ringmap`: a RingMapper correlations file
- `ss`: a structure diagram file
- `pairprob`: a pairing probabilities file. Percentage values will be calculated by RNAvigate.
- `pdb`: a PDB atomic coordinates file

In [ ]:

```
tpp = rnav.Sample(
    sample="TPP DMS-MaP",
    dmsmap="DMS_TPP_profile.txt",
    ringmap="DMS_TPP_rings.txt",
    ss="TPP-2GDI.nsd",
    pairprob="TPP-dms-bp.dp",
    pdb={"pdb":"2gdi.pdb", "chain":"X"})
```

## Display normalized ShapeMapper2 profile¶

In [ ]:

```
plot = rnav.plot_profile(
    samples=[tpp],
    sequence="ss",
    profile="dmsmap"
    )
plot.set_figure_size(height_ax_in=1)
plot.axes[0,0].get_xaxis().set_visible(False)
plot.axes[0,0].get_yaxis().set_visible(False)
# plot.save("tpp_profile.svg")
```

## Compare modified and unmodified mutation rates¶

In [ ]:

```
plot = rnav.plot_skyline(
    samples=[tpp],
    sequence="ss",
    profile="dmsmap",
    columns=["Modified_rate", "Untreated_rate"])
plot.set_figure_size(height_ax_in=1)
plot.axes[0,0].get_xaxis().set_visible(False)
plot.axes[0,0].get_yaxis().set_visible(False)
plot.axes[0,0].get_legend().set_visible(False)
# plot.save("tpp_skyline.svg")
```

## Display secondary structure and pairing probabilities as arc plots¶

In [ ]:

```
plot = rnav.plot_arcs(
    samples=[tpp],
    sequence="ss",
    structure="ss",
    profile="dmsmap",
    interactions="pairprob",
    title=False,
    seqbar=False,
    plot_error=False,
    profile_scale_factor=8)
plot.axes[0,0].get_xaxis().set_visible(False)
# plot.save("tpp_arcs.svg")
```

## Display secondary structure and shape reactivity as circle plots¶

In [ ]:

```
plot = rnav.plot_circle(
    [tpp],
    sequence="ss",
    profile="dmsmap",
    colors={"sequence": "profile"},
    structure="ss",
    )
plot.axes[0, 0].get_xaxis().set_visible(False)
# plot.save("tpp_circle.svg")
```

## Display secondary structure drawing with nucleotides colored by reactivity¶

In [ ]:

```
plot = rnav.plot_ss(
    [tpp],
    structure="ss",
    profile="dmsmap",
    colors={
        "structure": "profile",
        "nucleotides": "profile",
        },
    bp_style="conventional")
# plot.save("tpp_ss.svg")
```

## Display filtered RINGs on 3D structure¶

Here, we calculate mutual information, and add it to the ringmap data for filtering. See the notebook for figure 4 for a full explaination.

In [ ]:

```
tpp.data["ringmap"].data.eval("MI = Statistic / (2 * Mod_Depth)", inplace=True)

plot = rnav.plot_mol(
    [tpp],
    structure="pdb",
    interactions={
        "interactions":"ringmap",
        "structure": "ss",
        "positive_only": True,
        "Statistic_ge": 23,
        "min_cd": 6,
        "ss_only":True,
        "Zij_ge":2,
        "MI_ge": 0.000025
        },
    width=800,
    height=800,
    background_alpha=0,
    orientation=[17.15,9.70,-0.02,-74.23,-0.79,-0.35,-0.18,0.46],
    hide_cylinders=True,
    colors="grey",
    title=False)
```

You appear to be running in JupyterLab (or JavaScript failed to load for some other reason). You need to install the 3dmol extension:   
jupyter labextension install jupyterlab\_3dmol

In [ ]:

```
# plot.save()
```
