# Supplementary material for: RNAvigate: efficient exploration of RNA chemical probing datasets
Source: Nucleic Acids Res. 2024 Feb 13;52(5):2231–41. doi: 10.1093/nar/gkae089 (PMC10954457; doi:10.1093/nar/gkae089)
Supplement: gkae089_Supplemental_Files [file gkae089_supplemental_files.zip › RNAvigate_SI_static_notebooks.zip!/RNAvigate_SI_static_notebooks/F6_compare_SARS2.html]

F6\_compare\_SARS2


# Comparison of chemical probing methods¶

Here we compare the results of four studies that reported full-length SARS-CoV-2 secondary structures based on in-cell structure probing data.

In short, cells infected with SARS-CoV-2 were probed with a structure probing reagent. Then, sites of chemical adducts were encoded in cDNA libaries and sequenced. Chemical reactivity is quantified, per-nucleotide, using a bioinformatic pipeline. Structure prediction is performed using chemical reactivity as constraints. All four studies differed in the details of each of these steps. Refer to Table S1 for the differences between these experiments.

For each publication, per-nucleotide reactivities and secondary structure models were downloaded from the original publications with one exception. The full-length structure model from Sun et al. was not included in the original publication, but was found in the supplemental data from Lan et al.

In addition, we performed our own structure modelling, using RNAstructure Fold, of the first 8000 nucleotides of SARS-CoV-2, with a max pairing distance of 300 (`--md 300`) and without chemical probing data.

Our *de novo* structure model:

- Files and commands (RNAvigate\_figures/interactive\_notebooks/figure\_6/denovo\_model)
- Structure model file

Manfredonia et al. 2020 link

- Data set website link
- Reactivity data file download
- Structure model file download

Sun et al. 2021 link

- Reactivity data file download

Lan et al. 2022 link

- Structure model file download
- Reactivity data file download
- Supplemental data file containing Sun et al. structure model file download

Huston et al. 2021 link

- Github repo link
- Reactivity data file download
- Structure model file download

## Import RNAvigate¶

The first step in this notebook is to import the necessary Python modules: RNAvigate and Pandas. Pandas is needed to load Excel files into RNAvigate.

In [ ]:

```
import rnavigate as rnav
import pandas as pd
```

## Define samples and provide input file names¶

Next, data files are loaded into RNAvigate samples and assigned to data keywords.

- `shapemap` stores SHAPE-MaP, icSHAPE, or DMS-MaPseq reactivities, depending on the study.
- `ss` stores the secondary structure model from each study.

We create one sample for each study (`lan`, `huston`, `manfredonia`, and `sun`),
and one sample to hold our no-data MFE model (`de_novo`).

RNAvigate does not natively accept Excel files. Instead, Pandas is used to create DataFrames, which are accepted, from the Excel files. This requires only one extra python function `pandas.read_excel`.

In [ ]:

```
de_novo = rnav.Sample(
    sample="No data MFE",
    ss="./denovo_model/sars_1_8000_md300.ct")

huston = rnav.Sample(
    sample="Huston et al.",
    shapemap="./Huston_etal_2021/SARS-CoV-2_SHAPE_Reactivity.map",
    ss="./Huston_etal_2021/SARS-CoV-2_Full_Length_Secondary_Structure_Map.ct")

manfredonia = rnav.Sample(
     sample="Manfredonia et al.",
     shapemap={
         "shapemap_rnaframework": "./Manfredonia_etal_2020/SHAPE_invivo/SHAPE_invivo.xml"
         },
     ss="./Manfredonia_etal_2020/SHAPE_invivo/SARS-CoV-2.db")

lan_profile_df = pd.read_excel(
    io="./Lan_etal_2022/population_reactivities.xlsx",
    sheet_name="mus",
    usecols=[0, 1, 3],
    names=["Nucleotide", "Sequence", "Norm_profile"])

lan = rnav.Sample(
    sample="Lan et al.",
    shapemap=lan_profile_df,
    ss="./Lan_etal_2022/vero.ct")

sun_profile_df = pd.read_excel(
    io="./Sun_etal_2021/mmc2.xlsx",
    sheet_name="SARS2-invivo",
    usecols=[0, 1, 2],
    names=["Nucleotide", "Sequence", "Norm_profile"],
    dtype={
        "Nucleotide": "Int32",
        "Sequence": "string",
        "Norm_profile": "float32"}
    )

sun_ss_df = pd.read_excel(
    io="./Lan_etal_2022/all_figure_data.xlsx",
    sheet_name="Figure S5",
    usecols=[0, 13],
    names=["Nucleotide", "Pair"],
    nrows=21289,
    skiprows=5,
    dtype={
        "Nucleotide": "Int32",
        "Pair": "Int32"})

pairs_list = [(nt, pair_nt) for _, (nt, pair_nt) in sun_ss_df.iterrows()]

sun = rnav.Sample(
    sample="Sun et al.",
    shapemap=sun_profile_df,
    ss={'ss_pairs': pairs_list, 'sequence': 'shapemap'}
    )
```

## Change sequences to uppercase RNA alphabet¶

Some of the provided files specify a DNA alphabet and may contain lowercase letters.
Here, all associated sequences are converted to an uppercase RNA alphabet.

In [ ]:

```
# Make sure that all sequences are RNA alphabet and uppercase
for sample in [lan, huston, manfredonia, sun, de_novo]:
    for data_keyword in sample.data.keys():
        sample.get_data(data_keyword).normalize_sequence(
            t_or_u='U',
            uppercase=True,
            )
```

## Normalizing raw reactivities from Lan et al.¶

Lan et al. profile data were provided as raw mutation rates. Here, DMS data are renormalized according to their methods:

From Lan et al.:

> Normalizing the DMS reactivities
>
> For purposes of folding RNA structures using DMS reactivity constraints, DMS
> reactivities were normalized to a scale of 0–1 as follows. The median was
> computed among the top 5% of DMS reactivities (except where a different
> percentage is specified). All DMS reactivities were divided by this median to
> compute the normalized reactivities. Normalized reactivities greater than 1.0
> were winsorized by setting them to 1.0.

Seen in the resulting output of this code block, RNAvigate produces a warning that Lan et al. (shapemap) data are missing error rates. In this case, RNAvigate performs normalization without propagation of error.

In [ ]:

```
# Perform normalization of raw reactivities from Lan et al.
lan.get_data('shapemap').normalize(
    norm_method='percentiles',       # normalize based on the the median or mean of a percentile range
    nt_groups=['A','C'],             # normalize A and C together, do not include U or G
    median_or_mean="median",         # divide data by the median of values between 95th and 100th percentile
    lower_bound=95,
    upper_bound=100,
    )
lan.get_data('shapemap').winsorize(
    column="Norm_profile",           # winsorize the Normalized profile from the previous step
    lower_bound=None,                # do not apply a lower bound
    upper_bound=1.0,                   # for values above 1, set to 1
    )
```

```
Warning: Lan et al. (shapemap) missing expected error column
```

## Check sequence alignments¶

We don't know if the sequences being used differ positionally from each other.
RNAvigate can perform its own sequence alignment but it is computationally expensive.
We can also use RNAvigate's SequenceChecker to write sequences to a fasta file, then use an external sequence aligner.
After running this code, the "sequences.fa" file is input into ClustalOmega (link) to obtain a multiple sequence alignment.

ClustalOmega options:

- RNA sequence input format
- Pearson/FASTA output format
- otherwise default settings

Next, the sequence alignment file from the previous step is loaded into RNAvigate using `rnav.data.set_multiple_sequence_alignment`.
Whenever data is compared between these samples, they are first positionally aligned using this alignment.

In [ ]:

```
seq_check = rnav.analysis.SequenceChecker(samples=[lan, huston, manfredonia, sun, de_novo])
seq_check.write_fasta('sequences.fa')
```

In [ ]:

```
base_seq = rnav.data.set_multiple_sequence_alignment('alignments.fa', set_pairwise=True)
```

## Compare normalized reactivities between experiments¶

In order to determine the extent of agreement between experimental reactivities,
RNAvigate is used to plot sample vs. sample reactivies and to compute Pearson coefficients.
Here, a kernel density plot is used, instead of the default scatter plot,
which allows better visualization for large data sets.
We apply the region argument to limit our analysis to the first 7kb of ORF1a.

In [ ]:

```
plot = rnav.plot_linreg(
    samples=[lan, huston, manfredonia, sun],
    profile="shapemap",
    scale='linear',
    region=[266, 7265],
    regression="pearson",
    kde=True,
    colorbars=False,
    )
for row in plot.axes:
    for ax in row:
        ax.set(
            xlim=(-0.1, 2.6),
            ylim=(-0.1, 2.6),
            xticks=[0, 0.5, 1.0, 1.5, 2.0, 2.5],
            yticks=[0, 0.5, 1.0, 1.5, 2.0, 2.5],
        )
plot.save("pairwise_regression.svg")
```

## Compare structure models¶

Below, we create new RNAvigate interactions data `structure_comparison` which contains information on every base-pair modelled in any of the four studies, including which models and how many models contain that base-pair.

Using this data, we compute the percentage of base pairs in the first 7 kb of ORF1a that are predicted by 1, 2, 3 or 4 studies. If a base-pair is predicted in multiple studies, it is included that many times.

The plot below is created from RNAvigate data objects, but uses matplotlib to create the pie chart.

In [ ]:

```
import matplotlib.pyplot as plt

structure_comparison = rnav.data.StructureCompareMany(
    input_data=[
        lan.get_data("ss"),
        huston.get_data("ss"),
        manfredonia.get_data("ss"),
        sun.get_data("ss")
        ],
    sequence=lan.get_data("ss"),
    )

df = structure_comparison.data
orf1 = df.eval("i >= 266 & j <= 7265")
num_structures = df.loc[orf1, "Num_structures"] + 1
percentages = [sum(num_structures == i)*(i) / sum(orf1) for i in [1, 2, 3, 4]]

fig, ax = plt.subplots(1, figsize=(2, 2))
ax.pie(
    x=percentages,
    labels=["1 study", "2", "3", "all studies"],
    colors=structure_comparison._metric["cmap"],
    startangle=90,
    autopct='%1.0f%%');

# plt.savefig("pie_chart.svg")
```

# Compare no-data MFE model to study models¶

Here we compute two statistics to compare our no-data MFE model to the 4 study models:

- how many base-pairs in the no-data MFE model appear in zero studies
- how many consensus base-pairs (in 3-4 studies) are not in the MFE model

In [ ]:

```
region = list(range(266, 7266))
denovo_ss = de_novo.get_data("ss")

denovo_int = denovo_ss.as_interactions()
denovo_int.mask_on_position(isolate=region)
denovo_int = denovo_int.copy(apply_filter=True)

structure_comparison.reset_mask()
structure_comparison.mask_on_position(isolate=region)
studies = structure_comparison.copy(apply_filter=True)

in_denovo = studies.filter(
    structure=denovo_ss,
    paired_only=True,
    isolate_nts=region
    )
in_3_or_4 = studies.mask_on_values(Num_structures_ge=3)

consensus = sum(in_3_or_4)
consensus_in_denovo = sum(in_3_or_4 & in_denovo)
consensus_not_in_denovo = consensus - consensus_in_denovo
denovo_total = len(denovo_int.data)
denovo_not_in_studies = denovo_total - sum(in_denovo)

print(f"modelled de novo, not in any study: {denovo_not_in_studies} / {denovo_total} = {denovo_not_in_studies / denovo_total:.2%}")
print(f"consensus pairs not predicted de novo: {consensus_not_in_denovo} / {consensus} = {consensus_not_in_denovo / consensus:.2%}")
```

```
modelled de novo, not in any study: 552 / 2257 = 24.46%
consensus pairs not predicted de novo: 143 / 1234 = 11.59%
```

## Visualize structure models¶

To visualize these structure models, we use `rnav.plot_arcs()` using the `structure_comparison` data created early and our no-data MFE model.
`structure_comparison` is displayed on arc plots with each base-pair colored by how many models predict it.
To limit the size of these plots, we only visualize nucleotides 1000-4000 using the `region` argument.

Each group choose a different value for the max pairing distance parameter provided to the folding algorithm, which limits the similarity seen between structure models.
These limits are displayed on this plot using the Matplotlib interface of the RNAvigate plot.
Each dashed line represents the maximum height of an arc reported by one of the studies.

We will create additional plots to enlarge areas which are distinctly different between MFE and structure probing models. Here, we draw boxes around these regions.

In [ ]:

```
start, end = 1000, 4000
plot = rnav.plot_arcs(
    samples=[lan],
    labels=["All samples"],
    sequence=structure_comparison,
    structure=de_novo.get_data("ss"),
    interactions=structure_comparison,
    panels={"interactions": "top",
            "structure": "bottom"},
    region=[start, end],
    seqbar=False,
    colorbars=False,
    nt_ticks=(500, 100),
    )

# custom lines and boxes
ax = plot.axes[0, 0]
ax.set(ylim=(-305, 305))
# Draw boxes around the zoom-in regions
for x1, x2 in [[1635, 1960], [2810, 3112]]:
    y1, y2 = -80, 60
    ax.plot(
        [x1, x2, x2, x1, x1],
        [y1, y1, y2, y2, y1],
        color="black", ls='--', lw=1
        )
# Draw dashed lines for maximum pairing distances
for pairing_distance in [350, 300, 500, 600]:
    ax.plot(
        [start, end],
        [pairing_distance/2, pairing_distance/2],
        color="grey", ls="--",
        )
# set the figure size (1000 nts == 3 inches)
plot.set_figure_size(height_ax_rel=0.003, width_ax_rel=0.003)

# plot.save(f"ss_compare_{start}_{end}.svg")

cb_plot = plot.plot_colorbars()
# cb_plot.save("ss_compare_colorbar.svg")
```

## Zoom-in of regions where the MFE model diverges from consensus base-pairs¶

In [ ]:

```
for start, end in [[1635, 1960], [2810, 3112]]:
    plot = rnav.plot_arcs(
        samples=[lan],
        labels=["All samples"],
        sequence=structure_comparison,
        structure=de_novo.get_data("ss"),
        interactions=structure_comparison,
        panels={"interactions": "top",
                "structure": "bottom"},
        region=[start, end],
        seqbar=False,
        colorbars=False,
        nt_ticks=(100, 50),
        )
    ax = plot.axes[0, 0]
    # restrict the y-axis
    ax.set(ylim=(-80, 60))
    # set the figure size (1000 nt == 8 inches)
    plot.set_figure_size(height_ax_rel=0.008, width_ax_rel=0.008)
    # plot.save(f"ss_compare_{start}_{end}.svg")
```
