# Supplementary material for: RNAvigate: efficient exploration of RNA chemical probing datasets
Source: Nucleic Acids Res. 2024 Feb 13;52(5):2231–41. doi: 10.1093/nar/gkae089 (PMC10954457; doi:10.1093/nar/gkae089)
Supplement: gkae089_Supplemental_Files [file gkae089_supplemental_files.zip › RNAvigate_SI_static_notebooks.zip!/RNAvigate_SI_static_notebooks/F4_tr-folding_RNaseP.html]

F4\_tr-folding\_RNaseP


# Time-resolved probing of RNase P¶

This is a recreation of figures and analysis from Figure 3 of Ehrhardt & Weeks 2020.
pdf

Raw sequencing reads, secondary structure diagram, and three-dimensional structure were obtained from the authors.
Raw sequencing reads were analyzed using ShapeMapper v2.1.5 (with `--amplicon` and `--output-parsed-mutations` parameters).
The resulting parsed mutation strings files (`_parsed.mut`) were analyzed further using RingMapper (default parameters).
We focus on datasets at 0 (no Mg^2+^), 30 and 1200 seconds.
There are thus eight required files for this analysis, included in this directory:

- 3dhsCrystal\_PlusLoops.pdb
- RC\_CRYSTAL\_STRUCTURE.xrna
- RC0\_rnasep\_profile.txt
- RC0\_rnasep\_rings.txt
- RC30\_rnasep\_profile.txt
- RC30\_rnasep\_rings.txt
- RC1200\_rnasep\_profile.txt
- RC1200\_rnasep\_rings.txt

## Import RNAvigate¶

In [ ]:

```
import rnavigate as rnav
```

## Define the experimental samples and provide input file names¶

Here we have 3 samples, TMO-treated RNase P construct:

1. in the absence of Mg^2+^ (No Mg^2+^)
2. folded with Mg^2+^ for 30 seconds (partially folded)
3. folded with Mg^2+^ for 1200 seconds (fully folded)

Inputs common to all samples:

- `ss`: a structure drawing file based on the crystal structure
- `pdb`: 3D structure based on 3DHS as a .pdb file
- One annotation for each of the regions defined in the original publication,
  which are involved in the largest changes between time-points:
  - `catalytic_core`, `P5`, `P2`, `loop_loop`

Inputs unique to each sample:

- `sample`: an arbitrary string used as a label on plots
- `dmsmap`: a ShapeMapper2 profile.txt
  - When using `dmsmap` instead of `shapemap` as the keyword, data are automatically rescaled using the same convention as PairMapper (manuscript citation 27).
- `ringmap`: a RingMapper correlations file

In [ ]:

```
common_data = rnav.Sample(
    sample='',
    ss="RC_CRYSTAL_STRUCTURE.xrna",
    pdb={"pdb": "3dhsCrystal_PlusLoops.pdb",
         "chain":"A"},
    catalytic_core={
        "spans": [[236, 240], [181, 188], [45, 47], [49, 49], [127, 128]],
        "sequence": "ss",
        "color": "DarkOrchid",
        "name": "catalytic core"},
    P5={
        "spans": [[58, 62], [101, 105]],
        "sequence": "ss",
        "color": "orange",
        "name": "P5 helix"},
    P2={
        "spans": [[189, 195], [15, 21]],
        "sequence": "ss",
        "color": "DodgerBlue",
        "name": "P2 helix"},
    loop_loop={
        "spans": [[70, 75], [141, 146]],
        "sequence": "ss",
        "color": "red",
        "name": "L5.1 - L15.1"},
    )

rc0 = rnav.Sample(
    sample="No Mg$^{2+}$",
    inherit=common_data,
    dmsmap="RC0_rnasep_profile.txt",
    ringmap="RC0_rnasep_rings.txt")

rc30 = rnav.Sample(
    sample="partially folded",
    inherit=common_data,
    dmsmap="RC30_rnasep_profile.txt",
    ringmap="RC30_rnasep_rings.txt")

rc1200 = rnav.Sample(
    sample="fully folded",
    inherit=common_data,
    dmsmap="RC1200_rnasep_profile.txt",
    ringmap="RC1200_rnasep_rings.txt")

sample_list = [rc0, rc30, rc1200]
```

## Additional information for each sample¶

The raw sequencing reads used in this analysis differ slightly from the original
publication in that they are sequenced to much higher read depth. This higher
read depth requires a slightly different filtering scheme. Instead of filtering
for $G\_{apc}$, which is dependent on read depth, we will filter using mutual
information (MI) which is directly related to $G\_{apc}$, but does not depend on
read depth.

$$ MI = \frac{G\_{apc}}{2 \* depth} \\ $$

Below, we calculate this value and add it to the "ringmap" dataframe for each
sample, so that we can use it later to filter RINGs.

In [ ]:

```
for sample in sample_list:
    sample.data["ringmap"].data.eval("MI = 0.5 * Statistic / Mod_Depth", inplace=True)
```

## Custom color list¶

We will use the annotations below to highlight important regions and also want
to color individual nucleotides by region. We will make a custom list of
colors, one color for each nucleotide in RNase P.

In [ ]:

```
annotations_list = ["catalytic_core", "P5", "P2", "loop_loop"]
color_list, cmap = common_data.get_data("ss").get_colors_from_annotations(
    annotations=common_data.get_data(annotations_list)
    )

plot = rnav.plots.ColorBar(1)
plot.plot_data(cmap)
plot.set_figure_size(width_ax_in=4)
```

## Display a detailed secondary structure drawing¶

Here, we make a structure diagram that includes important details, as a
reference for the more data-rich plots we will make later.

In [ ]:

```
plot = rnav.plot_ss(
    samples=[rc0],
    structure="ss",                   # make a secondary structure diagram
    colors={'sequence': color_list},  # use annotations colors
    annotations=annotations_list,     # highlight our important regions
    bp_style="conventional",          # use Westoff-Leonitis base-pairing conventions
)

ax = plot.axes[0,0]
# add these text labels to the plot axis
# ax.text(x position, y position, "displayed text")
ax.text(0.2, 1.5, "catalytic\ncore", color="DarkOrchid")
ax.text(-6, 7.5, 'P1')
ax.text(-6, -2.5, 'P4')
ax.text(-6, -8.5, 'P5', color='orange')
ax.text(-8.5, -8.5, 'P7')
ax.text(0.1, -8.5, 'P15')
ax.text(-6.5, -12.5, 'P5.1')
ax.text(4.5, 15, 'L19')
ax.text(8, 7.5, 'P19')
ax.text(8, 2, 'P2', color='DodgerBlue')
ax.text(8, -4.5, 'P3')
ax.text(4.3, -5.5, 'P15.2')
ax.text(4.5, -10.5, 'P15.1')
ax.text(3, -17, 'L15.1', color='red')
ax.text(-4.5, -17, 'L5.1', color='red');

# plot.save("rnasep_big_ss.svg")
```

## Display filtered RINGs on secondary structure drawing¶

Next, for each of the samples, we plot the RING-MaP interactions in green on
the secondary structure.

Note that the RNA sequence used in the experimental data contains structure
cassette sequences at the 5' and 3' ends, while the secondary structure and PDB
structure do not. When RNAvigate plots data on a structure, a sequence
alignment is performed to place the data in the correct position.

In [ ]:

```
temp_settings = {
    'ss': {
        'interactions': {
            'linewidth': 8,
            'alpha': 1},
        'nucleotides': {
            's': 6**2},
        'structure': {
            'zorder': 40,
            'linewidth': 6},
        'basepairs': {
            'zorder': 39}
    }}
with rnav.styles.Settings(temp_settings):
    plot = rnav.plot_ss(
        samples=sample_list,           # Make one structure diagram for each sample in our list
        structure="ss",
        interactions={
            'interactions': "ringmap", # show ringmap interactions
            'structure': 'ss',         # use the "ss" structure drawing to filter by structural features
            'MI_ge': 0.00025,          # Only include RINGs with MI value greater than or equal to 0.00025
            'Statistic_ge':23,         # Only include RINGs with Gapc value greater than or equal to 23
            'min_cd': 6,               # Only include RINGs with contact distance of at least 5
            'positive_only': True,     # Only include RINGs with a positive correlation
            'cmap':'limegreen',         # Set the color of all rings to "limegreen"
            },
        bp_style="line",               # represent base-pairs with solid lines
        colors={
            'structure': color_list,   # Apply custom color list to the backbone of the structure
            'nucleotides': color_list,
            },
        )
```

## Color RINGs from previous plot by origination time-point¶

The above plot is nice, but we would like to convey additional information.
Certain RINGs are newly formed at each time point, and others are carried over
from a previous time point. We will color these differently. The code below
includes some advanced manipulations of the underlying dataframes to acheive
this effect.

In [ ]:

```
# retreive the RING-MaP DataFrame for each sample
s1 = rc0.data['ringmap'].data
s2 = rc30.data['ringmap'].data
s3 = rc1200.data['ringmap'].data
# get i_offset and j_offset values as 2D numpy array
a = s1[['i', 'j']].values
b = s2[['i', 'j']].values
c = s3[['i', 'j']].values
# determine which rings are newly formed and store result in the dataframe
# is there 'any' row in a that completely matches all values of a row in 'b'
s1['new_rings'] = s1['mask']
s2['new_rings'] = ~(b[:, None] == a[s1['mask']]).all(-1).any(-1)
s3['new_rings'] = ~(c[:, None] == b[s2['mask']]).all(-1).any(-1)

with rnav.styles.Settings(temp_settings):
    plot = rnav.plot_ss(
        samples=sample_list,
        structure="ss",
        interactions={
            'interactions': 'ringmap',
            'structure': 'ss',
            'MI_ge': 0.00025,
            'Statistic_ge':23,
            'min_cd': 6,
            'positive_only': True,
            'cmap':['khaki', 'limegreen'],
            'metric': 'new_rings',
            'normalization': 'none'},
        bp_style='line',
        colors={
            'structure': color_list,
            'nucleotides': color_list},
        colorbars=False
        )

# add text labels for colored rings
plot.axes[0, 0].text(4, 8, 'newly formed\nin current\ntime point', color='limegreen', fontsize=16, ha='right')
plot.axes[0, 1].text(4, 8, 'present in\nprevious\ntime point', color='khaki', fontsize=16, ha='right');

# save plot to file
# plot.save('rnasep_rings_ss.svg')
```

## Display RINGs density and regions of interest using heatmaps¶

Heatmaps are a good way to view very dense 2D data but can be hard to
interpret. The reader is helped here by highlighting regions which
illustrate time-dependent structural interactions.

Here, we will use the kernal density estimate ("kde") to simplify the
visualization, an improvement over the published figure.

In [ ]:

```
regions = [((225,250), (80,110)),     # ((x1, x2), (y1, y2))
           ((225,255), (175,200)),    # highlights interactions between region
           ((135,150), (60,85)),      # x1-x2 and y1-y2
           ((60,155), (10, 30))]

plot = rnav.plot_heatmap(
    samples=sample_list,              # 1 plot for each of three samples
    sequence="ss",                    # align all data to the secondary structure sequence
    structure='ss',                   # use nucleotide positions from 'ss' data (excludes the structure cassetes)
    interactions={
        'interactions': 'ringmap',    # Plot RING-MaP interactions
        'positive_only': True,        # remove negative correlations
        'cmap': 'Greens',             # use matplotlib 'Greens' colormap
        },
    regions=regions,                  # draw boxes around regions
    plot_type='kde')                  # plot RINGs density instead of individual RINGs

# adding labels to region boxes
ax = plot.axes[0,0]
arrowprops = {'arrowstyle':'-', 'color': 'black'}
ax.annotate('P4-P7', [75, 205])
ax.annotate('P4-P19', [187.5, 220], [200, 175], arrowprops=arrowprops)
ax.annotate('L5.1-L15.1', [90, 142.5], [140, 120], arrowprops=arrowprops)
ax.annotate('P2-P5,P7', [35, 75], [75, 50], arrowprops=arrowprops)

# adding labels for density estimation
ax = plot.axes[0, 1]
ax.annotate('more\ndense', [170, 175], [200, 150], arrowprops=arrowprops)
ax.annotate('less\ndense', [125, 130], [150, 100], arrowprops=arrowprops)

# save plots to files
# plot.save('rnasep_heatmaps.svg')
```

Out[ ]:

```
Text(150, 100, 'less\ndense')
```

## Display filtered RINGs on 3D structure¶

In [ ]:

```
plot = rnav.plot_mol(
    samples=[rc1200],
    structure="pdb",
    interactions={
        'interactions': 'ringmap',
        'structure': 'ss',
        'MI_ge': 0.000025,
        'Zij_ge':2,
        'ss_only': True,
        'Statistic_ge':23,
        'min_cd': 6,
        'positive_only': True,
        'cmap': 'limegreen'},
    colors=color_list,
    hide_cylinders=True,
    width=800,
    height=800,
    background_alpha=0,
    orientation=[-35.06,-98.79,-28.57,-245.66,0.14,0.52,0.62,-0.57],
    title=False
    )
```

You appear to be running in JupyterLab (or JavaScript failed to load for some other reason). You need to install the 3dmol extension:   
jupyter labextension install jupyterlab\_3dmol

In [ ]:

```
# plot.save()
```
