# Supplementary material for: RNAvigate: efficient exploration of RNA chemical probing datasets
Source: Nucleic Acids Res. 2024 Feb 13;52(5):2231–41. doi: 10.1093/nar/gkae089 (PMC10954457; doi:10.1093/nar/gkae089)
Supplement: gkae089_Supplemental_Files [file gkae089_supplemental_files.zip › RNAvigate_SI_static_notebooks.zip!/RNAvigate_SI_static_notebooks/F5_DANCE_add-riboswitch.html]

F5\_DANCE\_add-riboswitch


# DANCE-MaP analysis of the adenine riboswitch¶

Here we recreate the visualization from Figure 2C of Olson et al. 2022. pdf

In short, DMS-MaP was performed on an IVT construct of *Vibrio vulnificus* adenine riboswitch in the absence of adenine ligand. DANCE-MaP analysis recapitulates the known states and populations of this riboswitch and provides direct evidence for accepted secondary and tertiary structure interactions. These data are then used to accurately model each state of the ensemble.

DanceMapper post-analysis files are availabe through the GEO (GSE182552). The direct download link is: GSE182552\_RAW.tar. Extract this folder and the resulting folder, GSM5531266\_wt\_0\_deep\_danceoutput.tar.gz to obtain the Dancemapper output files. To predict structures from these data, we run `python ~/DanceMapper/foldClusters.py --bp wt_0_deep wt_0_deep-reactivities.txt wt_0_deep` which produces .ct files. These .ct files were loaded into StructureEditor, part of RNAstructure. With this software, structure cassettes were removed and a custom layout for each secondary structure was created to match the original publication (wt\_0\_deep-0.nsd and wt\_0\_deep-1.nsd). Finally, the adenine-bound crystal structure was retreived from the RCSB, here. These steps yield the 8 files needed for this analysis:

From RCSB:

- 4tzx.pdb

From foldClusters.py followed by StructureEditor:

- wt\_0\_deep-0.nsd
- wt\_0\_deep-1.nsd

From original publication:

- wt\_0\_deep-0-pairmap.txt
- wt\_0\_deep-1-pairmap.txt
- wt\_0\_deep-0-rings.txt
- wt\_0\_deep-rings.txt
- wt\_0\_deep-reactivities.txt

## Import RNAvigate¶

In [ ]:

```
import rnavigate as rnav
```

## Define experimental samples and provide input file names¶

The second step is to organize data into samples by providing RNAvigate with input file names.
Each input file is converted to a Python data structure and assigned to a data keyword.
These data keywords tell RNAvigate how to parse the data, and organize data consistently between samples.
Here, each sample is defined as a single component of the DANCE-MaP model.
The data keywords for each sample are as follows:

- `dancemap`: reactivities from the DanceMapper "reactivities.txt" output file.
- `pairmap`: PAIRs from the DanceMapper "pairmap.txt" output file.
- `ringmap`: RINGs from the DanceMapper "rings.txt" output file.
- `ss`: secondary structure drawing from the StructureEditor ".nsd" output file.
- `pdb`: structure from the "4TZX.pdb" file downloaded from the PDB.
- `SD`: annotation of the Shine-Delgarno sequence, defined manually.

Finally, we calculate mutual information scores to be used to filter RINGs.
See the Jupyter notebook for figure 4 for a full explaination.

In [ ]:

```
add_wt_0 = rnav.Sample(
    sample="Translation OFF (58%)",
    dancemap={
        "dancemap": "wt_0_deep-reactivities.txt",
        "component": 0},
    pairmap="wt_0_deep-0-pairmap.txt",
    ringmap='wt_0_deep-0-rings.txt',
    ss='wt_0_deep-0.nsd',
    SD={
        'spans': [[99, 105]],
        'sequence': 'ss',
        'color': 'DarkOrchid',
        'name': "Shine-Delgarno"
        },
    pdb={'pdb': '4tzx.pdb', 'chain': 'X'},
    )

add_wt_1 = rnav.Sample(
    sample="Translation ON (42%)",
    dancemap={
        "dancemap": "wt_0_deep-reactivities.txt",
        "component": 1},
    pairmap="wt_0_deep-1-pairmap.txt",
    ringmap='wt_0_deep-1-rings.txt',
    ss='wt_0_deep-1.nsd',
    SD={
        'spans': [[99, 105]],
        'sequence': 'ss',
        'color': 'DarkOrchid',
        'name': "Shine-Delgarno"
        },
    pdb={'pdb': '4tzx.pdb', 'chain': 'X'},
    )

samples = [add_wt_0, add_wt_1]

# for each component of Dance, compute RINGs mutual information scores
for sample in samples:
    sample.data["ringmap"].data.eval(
        "MI = Statistic / (2 * Mod_Depth)",
        inplace=True
        )
```

## Data positions with different sequences¶

Experimental and structural data with different underlying sequences or
reference positions are automatically aligned and repositioned for visual
comparison. These alignments work well when sequences are similar, but it
is a good idea to check this alignment with more divergent sequences.

These alignments can be checked visually using `rnav.plot_alignment()`. Top and
bottom axes indicate positional alignment. Grey bars indicate sequence matches.

In [ ]:

```
plot = rnav.plot_alignment(
    data1=(add_wt_1, "dancemap"),
    data2=(add_wt_1, "ss"),
    labels=["dancemap", "ss"])
plot = rnav.plot_alignment(
    data1=(add_wt_1, "dancemap"),
    data2=(add_wt_1, "pdb"),
    labels=["dancemap", "pdb"])
```

## Display RINGs, PAIRs, and structure model for each component as arc plots¶

Olson et. al. established filters for visualizing RING-MaP and PAIR-MaP data
that result from DANCE-MaP analysis. These filters are applied below with
`interactions.filter(...)`. We are using a modified version of these filters.

A RING correlation between nucleotides i and j is included if the correlation:

- is positive
- has G_apc_ above 23
- has a contact distance between i and j of at least 15
- is between single-stranded nucleotides
- has a mutual information score greater than 0.000025
- has a Z_ij_ greater than 2

PAIRs are included only if they agree with the predicted structure.

Then, `rnav.plot_arcs_multisample(...)` is used to create arc plots for each
DANCE component. Arguments are explained in-line below.

In [ ]:

```
ss_list = [sample.get_data("ss") for sample in samples]
for sample in samples:
    ss, ringmap, pairmap = sample.get_data(["ss", "ringmap", "pairmap"])
    ringmap.filter(
        structure=ss,
        positive_only=True,
        ss_only=True,
        MI_ge=0.000025,
        Zij_ge=2,
        Statistic_ge=23,
        )
    ringmap.mask_on_structure(ss_list, min_cd=15)
    pairmap.filter(structure=ss, paired_only=True)

plot = rnav.plot_arcs(
    samples=samples,                 # Make one plot per dance component
    sequence="ss",                   # align sequences to structure model
    structure="ss",                  # display base-pair arcs
    profile="dancemap",              # display dance map profile bar plot
    interactions={
        "interactions": "pairmap",   # Plot PAIR-MaP data
        "prefiltered": True},        # Preserve the filtering applied above
    interactions2={
        "interactions": "ringmap",   # Plot RING-MaP data (on bottom by default)
        "prefiltered":True},         # Preserve the filtering applied above
    panels={
        "structure": "top",          # structure arcs on top
        "interactions": "top",       # PAIRs arcs on top
        "interactions2": "bottom"    # RINGs arcs on bottom
        },
    profile_scale_factor=5,          # Scale profile bar height by 5x
    plot_error=False,                # Don't plot error bars
    plot_kwargs={"track_labels": False}
    )
# plot.save("ade_arcs.svg")
```

```
Warning: Translation OFF (58%) (dancemap) missing expected error column
Warning: Translation ON (42%) (dancemap) missing expected error column
```

## Display filtered RINGs on secondary structure drawings for each component¶

In [ ]:

```
temp_settings = {
    'ss': {
        'interactions': {
            'zorder': 0,
            'linewidth': 3
    }}}
with rnav.styles.Settings(temp_settings):
    plot = rnav.plot_ss(
        samples=samples,                 # plot structure diagram for each dance component
        structure='ss',                  # display these structure drawings
        profile='dancemap',              # load reactivities data for coloring
        annotations=['SD'],              # highlight the shine-dalgarno sequence
        interactions2={
            'interactions': 'pairmap',   # display PAIRs
            'prefiltered': True},        # maintain the dance filter
        interactions={
            'interactions': 'ringmap',   # display RINGs
            'prefiltered': True},        # maintain the dance filter applied earlier
        colors={'sequence': 'profile'},  # use dancemap reactivity colors applied to the sequence letters
        )
plot.get_ax(0).text(6, -6, 'SD')
plot.get_ax(1).text(3, -1, 'SD');
# plot.save('ade_ss.svg')
```

## Display filtered RINGs on 3D structure¶

RINGs are filtered according to secondary structure. This requires a positional
alignment as described above. These filtered RINGs are then repositioned again
to be displayed on the 3D structure below. This multiple repositioning of data
is a significant time-saving feature of RNAvigate.

In [ ]:

```
plot = rnav.plot_mol(
    samples=[add_wt_1],
    structure="pdb",                # make a 3D rendering of the ON state
    interactions={
        'interactions': 'ringmap',  # plot RING-MaP interactions
        'prefiltered': True},       # preserve the filtering applied above
    atom='DMS',                     # Draw RING cylinders between DMS-reactive atoms (N1 for A and C, N3 for U and G)
    colors='grey',                  # Color RNA grey
    width=800, height=800,          # change plot dimensions
    background_alpha=0,             # make background transparent
    orientation=[21.49, -19.50, 11.15, -70.90, -0.50, -0.84, -0.04, 0.22],
                                    # use this predetermined orientation
    title=False,                    # don't display the title
)
```

You appear to be running in JupyterLab (or JavaScript failed to load for some other reason). You need to install the 3dmol extension:   
jupyter labextension install jupyterlab\_3dmol

In [ ]:

```
# plot.save()
```
