# Supplementary material for: RNAvigate: efficient exploration of RNA chemical probing datasets
Source: Nucleic Acids Res. 2024 Feb 13;52(5):2231–41. doi: 10.1093/nar/gkae089 (PMC10954457; doi:10.1093/nar/gkae089)
Supplement: gkae089_Supplemental_Files [file gkae089_supplemental_files.zip › RNAvigate_SI_static_notebooks.zip!/RNAvigate_SI_static_notebooks/F3_lowSS_DENV.html]

F3\_lowSS\_DENV


# Low SHAPE/low Shannon entropy analysis of DENV2 genome¶

This is a recreation of an analysis in Figure 1 from Dethoff et al. 2018. Data files
were obtained from the authors. pdf

- DENV2\_EX\_1M7\_MAP.map
- DENV2\_EX-Diff\_BP-prob.dp
- DENV2\_EX-Diff\_MFE.ct

These files were originally produced by performing SHAPE-MaP, analyzing resulting sequencing
reads using ShapeMapper, and then producing a secondary structure model and base
pairing probabilities using SuperFold.

In this example, we will analyze SHAPE-MaP data from the Dengue 2 viral genome.
low SHAPE, low Shannon entropy (lowSS) regions in a long RNA can be used to
detect regions of well-defined structure. Functional elements tend to be
over-represented in these lowSS regions. These regions are defined by low SHAPE
reactivity, indicating low local nucleotide flexibility, and by low Shannon
entropy, a measure of likelihood to form alternative structures.

In [ ]:

```
import rnavigate as rnav
```

## Define the experimental sample and provide input file names¶

- `sample`: an arbitrary string that will serve as a label on plots
- `shapemap`: ShapeMapper2 profile.txt file
- `pairprob`: Superfold output file, .dp file providing basepairing probabilities
- `ct`: Superfold output file, .ct file providing an MFE structure

In [ ]:

```
denv = rnav.Sample(
    sample="DENV2 Genome",
    shapemap="DENV2_EX-1M7_MAP.map",
    pairprob="DENV2_EX-Diff_BP-prob.dp",
    ss="DENV2_EX-Diff_MFE.ct")
```

## Plot results of the low SHAPE, low Shannon entropy analysis¶

First, we will perform the lowSS analysis. By default, this step will produce a
full-length plot. However, DENV2 is 10732 nt, which is a bit unweildy, so we
will set `region=[1, 2000]` to limit the visualization to nucleotide positions 1 through 2000.

In [ ]:

```
analysis = rnav.analysis.LowSS(sample=denv)
plot = analysis.plot_lowss(region=[1,2000])

# plot.save("denv_lowss.svg")
```
